# The Sialoside-Binding Pocket of SARS-CoV-2 Spike Glycoprotein Structurally Resembles MERS-CoV

**DOI:** 10.3390/v12090909

**Published:** 2020-08-19

**Authors:** Mayanka Awasthi, Sahil Gulati, Debi P. Sarkar, Swasti Tiwari, Suneel Kateriya, Peeyush Ranjan, Santosh Kumar Verma

**Affiliations:** 1 Department of Cell Biology and Molecular Genetics, University of Maryland, College Park, MD 20742, USA; awasthi9@umd.edu; 2 Gatan Inc., Pleasanton, CA 94588, USA; s.gulati@case.edu; 3 Department of Biochemistry, University of Delhi South Campus, New Delhi 110021, India; dpsarkar59@gmail.com; 4 Department of Molecular Medicine & Biotechnology, Sanjay Gandhi Postgraduate Institute of Medical Sciences, Lucknow 226014, India; tiwari.pgi@gmail.com; 5 School of Biotechnology, Jawaharlal Nehru University, New Delhi 110067, India; skateriya@jnu.ac.in

**Keywords:** SARS-CoV-2, N-terminal domain, spike glycoprotein, MERS-CoV

## Abstract

COVID-19 novel coronavirus (CoV) disease caused by severe acquired respiratory syndrome (SARS)-CoV-2 manifests severe lethal respiratory illness in humans and has recently developed into a worldwide pandemic. The lack of effective treatment strategy and vaccines against the SARS-CoV-2 poses a threat to human health. An extremely high infection rate and multi-organ secondary infection within a short period of time makes this virus more deadly and challenging for therapeutic interventions. Despite high sequence similarity and utilization of common host-cell receptor, human angiotensin-converting enzyme-2 (ACE2) for virus entry, SARS-CoV-2 is much more infectious than SARS-CoV. Structure-based sequence comparison of the N-terminal domain (NTD) of the spike protein of Middle East respiratory syndrome (MERS)-CoV, SARS-CoV, and SARS-CoV-2 illustrate three divergent loop regions in SARS-CoV-2, which is reminiscent of MERS-CoV sialoside binding pockets. Comparative binding analysis with host sialosides revealed conformational flexibility of SARS-CoV-2 divergent loop regions to accommodate diverse glycan-rich sialosides. These key differences with SARS-CoV and similarity with MERS-CoV suggest an evolutionary adaptation of SARS-CoV-2 spike glycoprotein reciprocal interaction with host surface sialosides to infect host cells with wide tissue tropism.

## 1. Introduction

Multiple coronaviruses (CoV) are known to cause infection in humans of which β-coronavirus family members, namely severe acquired respiratory syndrome (SARS)-CoV, Middle East respiratory syndrome (MERS)-CoV, and recently SARS-CoV-2 outbreak is a serious threat to the public health and has infected over 7 million people including over 400,000 deaths worldwide with a latency period of 3–14 days [1]. Recently, the World Health Organization (WHO) officially declared the COVID-19 novel coronavirus disease (caused by SARS-CoV-2) a global pandemic. SARS-CoV-2 is a positive-strand RNA virus and like SARS-CoV and MERS-CoV, attacks the lower respiratory system, causing acute respiratory distress in the lungs [2]. Recent reports suggest that SARS-CoV-2 also targets multiple organ systems like the heart, liver, kidney, gastrointestinal system, and central nervous system [3,4,5]. The rapid infection rate and spread of SARS-CoV-2 in multiple organs could be understood well by studying the virus–host cell interaction and fusion. Spike glycoprotein of both SARS-CoV and SARS-CoV-2 interacts with the human angiotensin-converting enzyme-2 (ACE2) receptor of the host cell and mediates the adhesion of virus to the host cell. After a series of cleavages and conformational changes in the spike protein, SARS-CoV-2 fuses with the target cellular membrane [6,7]. X-ray crystallography and cryo-electron microscopy studies along with in vivo experiments confirm that SARS-CoV-2 entry in host cells is also achieved by the spike–ACE2 interaction [8]. However, a recent study suggests limited ACE2 expression in the human respiratory system [9]. Thus, the role of ACE2 as the sole receptor for SARS-CoV-2 infection needs to be revisited.

Several human coronaviruses including MERS-CoV, HCoV-OC43, and HCoV-HKU1 utilize alternative strategies to infect human host cells. While HCoV-OC43, and HCoV-HKU1 use host sialosides as the sole receptor to infect host cells, MERS-CoV utilizes a dual-receptor strategy using both protein and sialoside receptors to attach and infect host cells [10]. To understand the mechanism underlying the high infectivity of SARS-CoV-2 and its ability to use human sialosides as an alternate receptor, we performed a detailed comparative structural analysis of the N-terminal domain (NTD) of SARS-CoV-2 with that of MERS-CoV and SARS-CoV. Interestingly, the primary sequence analysis of the spike glycoprotein of SARS-CoV-2 revealed three divergent loop regions in the NTD region. Further analysis revealed the key involvement of the divergent loop regions to form a potential sialoside-binding pocket in the NTD of SARS-CoV-2 that appears to be reminiscent of the MERS-CoV sialoside-binding pocket [11]. Recent studies have also reported the presence of these divergent loop regions, but their spatial localization and potential to engage sialosides or other small molecules is not yet established [12,13]. Here we used in silico docking algorithms and molecular dynamic simulations to test the binding potential of this SARS-CoV-2 NTD pocket with different sialosides. Remarkably, the dynamic nature of the β14-β15 loop, which is one of the three divergent loop regions, potentiates the binding of SARS-CoV-2 NTD with diverse and larger sialosides. Our result suggests that SARS-CoV-2 NTD is similar to the NTD of MERS-CoV spike glycoprotein and can accommodate sialosides at neutral pH. Thus, our observation potentiates further studies involving the functional role of SARS-CoV-2-sialoside interaction during virus entry, tissue tropism, and in identifying novel therapeutic targets preventing such interaction. 

## 2. Methods

### 2.1. Phylogenetic Tree and Sequence Alignment

Phylogenetic analyses of the spike proteins of human coronaviruses were performed computationally by the neighbor-joining (NJ) method on MEGA-X [14] with 1000 bootstrap replicates. The same was also verified by the maximum likelihood ML method on MEGA-X. The topology was viewed by MEGA-X as well as with tree view and NJ plot [14]. Multiple sequence alignment of the NTD domains was performed with the Clustal W program [15].

### 2.2. Structure Prediction

The cryo-EM structures of SARS-CoV-2 (PDB ID: 6VXX) [8] and MERS-CoV (PDB ID: 6Q04) [11] spike glycoproteins were used as the starting point for all the studies [8]. The full-length model of SARS-CoV-2 spike glycoprotein (YP_009724390.1) was strongly biased on the crystal structure of SARS-CoV-2, while the extended loops were first modeled using MODELLER [16] version 9v24 using the model-loop procedure. The cryo-electron microscopy (cryo-EM) structures of SARS-CoV (PDB ID: 6ACC [17] and 5XLR [18]) and MERS-CoV (PDB ID: 6Q04 [11] and 5W9H [19]) spike glycoproteins were used to generate the full-length models of SARS-CoV and MERS-CoV, respectively. The 100 resulting models for each of the spike glycoprotein were ranked using discrete optimized protein energy (DOPE) [20] statistical potentials. The best scoring model was protonated, and energy minimized by using the Amber99Sb force field [21] and root mean square (RMS) gradient of 0.1 kcal mol^−1^ A^−2^. The minimized model was explicitly solvated by a spherical water box with cell borders placed at least 6 Å away from any protein atom using TIP3P water model. To neutralize the total charge, Na^+^/Cl^−^ counterions were added to a final salt concentration of 0.150 M. The solvated system was energy minimized by tethering all atoms by a harmonic potential to its starting coordinates with a 0.5 Å deviation. The molecular dynamics (MD) simulations were conducted with the Nosé–Poincaré–Anderson (NPA) algorithm and canonical ensemble (NVT). In the initialization step, the simulation was performed for 100 ps at 300 K, followed by a 100 ps equilibration step gradually increasing the temperature from 300 K to 310.15 K. Finally, the production step was carried out for 5000 ps using a 1 fs time step and applying harmonic positional constraints on protein by a force constant of 1 kcal mol^−1^ A^−2^ and non-rigid water molecules. During this step, the temperature was maintained at 310.15 K by a Langevin thermostat, the pressure at 1 atm by a Berendsen barostat, and a heavy atom tether standard deviation of 0.5 Å deviation. The stereochemical quality of all the protein models was assessed with the Molprobity server (Supplementary Table S1) [22].

### 2.3. Molecular Docking and Simulations

The molecular docking calculations were performed using the Lamarckian Genetic Algorithm implemented in Autodock 4.2.6 [23]. Initial ligand data files were generated from SMILES strings using the Grade Web Server full-length model of SARS-CoV-2 glycoprotein obtained after NPA simulations and the ligands were prepared using the Autodock Tools program [23]. A local search was performed using Autosite [24] to identify possible binding sites on the NTD of SARS-CoV-2 glycoprotein. A grid box of 80 × 80 × 80 Å was defined centered on the selected binding pocket to allow all sialosides to rotate freely. A minimum of ten docked poses were generated for each sialoside derivative and the best pose was selected based on the highest binding affinity. All sialoside-SARS-CoV-2 glycoprotein complexes were explicitly solvated by a spherical water box with cell borders placed at least 10 Å away from any protein or ligand atom. Na^+^/Cl^-^ counterions were added to a final salt concentration of 0.150 M to neutralize the total charge of the system. The solvated protein-ligand complexes were then protonated, and energy minimized by using an Amber12 [25] EHTforce field and RMS gradient of 0.1 kcal mol^−1^ A^−2^. Molecular dynamics simulations were carried out at 310.15 K, 1 atm pressure, and a heavy atom tether standard deviation of 0.5 Å. All systems were equilibrated for 50 ps followed by a 500 ps production step with a 2 fs time step and applying harmonic positional constraints of 1 kcal mol^−1^ A^−2^ and non-rigid water molecules.

## 3. Results and Discussion

### 3.1. The N-Terminal Domain (NTD) of the SARS-CoV-2 Spike Protein Contains Divergent Loop Regions That Are Structurally Analogous to MERS-CoV

**Figure 1:**
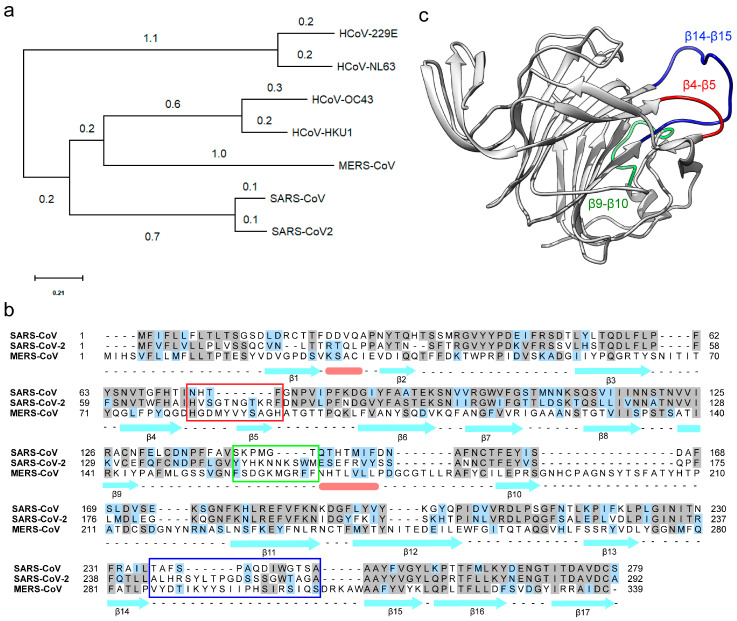
Phylogenetic, sequence and structural analysis of SARS-CoV-2 with close members of betacoronavirus family. (**a**) Evolutionary analysis of the spike protein of human-infecting coronaviruses. The phylogenetic tree was drawn by using the maximum likelihood method and scaled with branch lengths measured in the number of substitutions per site. (**b**) Sequence alignment of the N-terminal domain (NTD) of SARS-CoV, SARS-CoV-2, and MERS-CoV shows the divergent loop regions of an otherwise highly similar protein sequence. The β4-β5, β9-β10, and β14-β15 loop regions are highlighted by red, green, and blue box, respectively. The α-helices and β-strands are represented as pink bars and cyan arrows, respectively. (**c**) The tertiary structure of NTD of the spike protein of SARS-CoV-2. The β4-β5 (red) and β14-β15 (blue) loops are important components of the predicted sialoside-binding pocket of SARS-CoV-2 spike glycoprotein.

Receptor binding with the host cells is the initial step in virus infection, tissue tropism, and cell spread. Coronaviruses utilize a complex patterns of receptor recognition to infect diverse host cells. COVID-19 caused by SARS-CoV-2 has been a concern of increased global burden with high co-morbidity and mortality [26]. With a limited understanding of the diverse range of tissues targeted by the virus and its potential receptors, there is an immediate need to understand the SARS-CoV-2 entry mechanism and pathogenesis to develop effective therapeutics. Genome sequence-based phylogenetic analyses discuss the evolutionary origin of SARS-CoV-2 and its similarity with SARS-CoV [27]. Additional studies highlight the high degree of similarity between the receptor-binding domain (RBD) of SARS-CoV-2 and SARS-CoV, and their binding with the common human ACE-2 receptor [28]. Despite all these similarities, SARS-CoV-2 is more infectious than SARS-CoV [29]. MERS-CoV remains the only known human-infecting coronavirus that utilizes a dual-receptor strategy during infection. To investigate if SARS-CoV-2 could utilize similar reciprocal-receptor utilization, we analyzed the phyletic relatedness of the spike glycoproteins from coronaviruses that are known to infect humans. As expected, spike proteins of SARS-CoV and SARS-CoV-2 are highly similar and both groups are together in the same clade (Figure 1a). The closest spike glycoprotein to SARS clade is of the MERS-CoV, which suggests that MERS-CoV shares a higher similarity to SARS clade than other coronaviruses. Additionally, the spike protein of HCoV-229E and HCoV-NL63 form a separate clade (Figure 1a). Even though the spike proteins of HCoV-OC43 and MERS-CoV are distantly related, they both bind to host sialic acids as an alternate host-receptor during infection. To date, nothing is known about the interaction of the SARS clade with host sialic acid receptors.

Despite the 76% homology between spike-proteins, SARS-CoV-2 is more infectious than SARS-CoV, which suggests a possible structural or mechanistic difference [7]. One stark difference between their spike proteins is the presence of a furin-like cleavage site on the SARS-CoV-2 spike protein [30]. SARS-CoV-2 has 12 extra nucleotides encoding three amino acids upstream to the single Arg↓ cleavage site forming a PRRAR↓SV sequence, which is a canonical furin-like cleavage site [30]. The presence of this furin-like cleavage site in SARS-CoV-2 is predicted as a possible reason for its efficient spread as compared to the other beta coronaviruses [30]. Alternatively, by comparative sequence analysis of the NTD of spike protein, we identified three extended regions in SARS-CoV-2 and MERS-CoV (Figure 1b), but not in SARS-CoV (Figure 1b). To identify if the divergent loop region forms a functional module in the NTD of SARS-CoV-2, we modeled the full-length structure of SARS-CoV-2 spike glycoprotein strongly biased on the cryo-EM structure of SARS-CoV-2 spike protein (Figure 1c). The cryo-EM structures of SARS-CoV-2 spike protein display a well ordered β-strand rich NTD, RBD, and the core helical domain [8]. Owing to their flexibility, several β-β loops in the NTD of SARS-CoV-2 spike glycoprotein display almost no cryo-EM density even after *B*-factor sharpening. All unresolved β-β loops were modeled ab-initio and the model with the best DOPE score was further energy minimized and used for computational analyses as discussed in the methods section. Upon structural comparison of the modeled SARS-CoV-2 spike glycoprotein with SARS-CoV, we found a major difference concerning their β-β loop lengths. SARS-CoV-2 has larger, β4-β5, β9-β10, and β14-β15 loops in comparison to that of SARS-CoV (Figure 1b). The β14-β15 loop is particularly interesting owing to its length and flexibility due to the presence of interspersed glycine residues and a flanking poly-alanine region respectively (Figure 1b). The β14-β15 loop of SARS-CoV-2 is reminiscent of the β-hairpin loop (Ser126-Ile140) of MERS-CoV (Supplementary Figure S1a) [11]. The MERS-CoV β-hairpin loop features a similar long arm that forms critical electrostatic anchor points to host sialoside receptor engagement and stability [11].

### 3.2. The Divergent Loop Regions within SARS-CoV-2 NTD Forms a Sialoside-Binding Pocket

**Figure 2:**
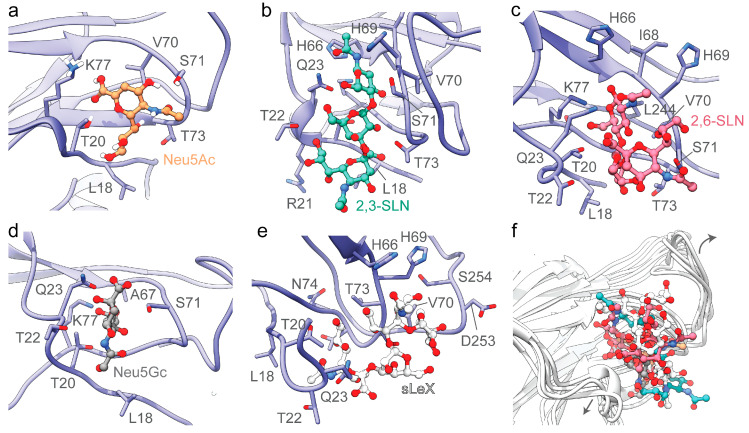
Binding between the docked sialoside derivatives and the NTD of SARS-CoV-2 glycoprotein. The key amino acid side chains surrounding (**a**) 5-N-acetyl neuraminic acid (Neu5Ac, orange), (**b**) α2,3-sialyl-N-acetyl-lactosamine (2,3-SLN, teal), (**c**) α2,6-sialyl-N-acetyl-lactosamine (2,6-SLN, pink) (**d**) 5-N-glycolyl neuraminic acid (Neu5Gc, dark grey), and (**e**) sialyl Lewis^X^ (sLeX, light grey) are shown. (**f**) The flexibility of β14-β15 loop (Leu244-Gly261) enables binding of a wide variety of sialosides. The NTD of SARS-CoV-2 glycoprotein is shown as purple ribbons and sialoside derivatives are shown as ball and stick models. The amino acids side chains interacting with sialosides are shown as purple sticks.

**Figure 3:**
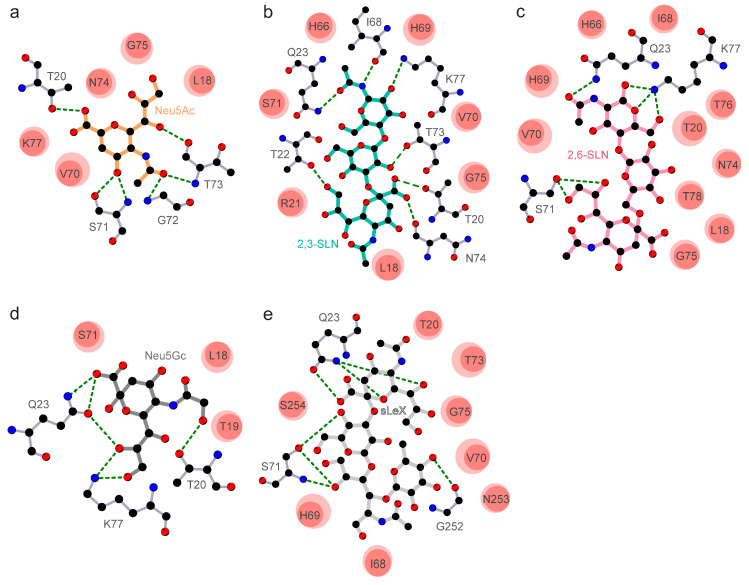
Protein-ligand interactions between the NTD of SARS-CoV-2 glycoprotein and (**a**) 5-N-acetyl neuraminic acid (Neu5Ac, orange), (**b**) α2,3-sialyl-N-acetyl-lactosamine (2,3-SLN, teal), (**c**) α2,6-sialyl-N-acetyl-lactosamine (2,6-SLN, pink), (**d**) 5-N-glycolyl neuraminic acid (Neu5Gc, dark grey) and (**e**) sialyl Lewis^X^ (sLeX, light grey) are shown. Dashed green lines show electrostatic interactions formed between the SARS-CoV-2 glycoprotein amino acid residues and the ligand. Hydrophobic contacts are shown as filled circles, where the orientation and size of the opaque ellipsoid mark the directionality and strength of hydrophobic interactions. In all panels, nitrogen, carbon, and oxygen atoms are colored blue, black, and red, respectively.

Despite the presence of the RBD domain that binds to host cell receptors, several coronaviruses utilize sugar-binding receptors as an alternative mode to bind and infect host cells. Such a dual-receptor binding mechanism allows enhanced infection and host-cell tropism [31]. To test the capacity of the SARS-CoV-2 spike protein to engage host-cell sialic acid receptors, we selectively docked 5-N-acetyl neuraminic acid (Neu5Ac), α2,3-sialyl-N-acetyl-lactosamine (2,3-SLN), α2,6-sialyl-N-acetyl-lactosamine (2,6-SLN), 5-N-glycolyl neuraminic acid (Neu5Gc), and sialyl Lewis^X^ (sLeX) (Figure 2) on to the modeled structure of SARS-CoV-2 NTD. The selected sialosides represent a large family of more than 500 human sialosides and have been previously shown to bind with the S1A domain of MERS-CoV [11]. The amino acid residues Leu18-Gln23, His66-Thr78 of the β4-β5 loop, and Gly252-Ser254 of β14-β15 loop forms the sialoside-binding site in SARS-CoV-2 spike protein (Figure 2 and Figure 3). Recently, Milanetti et al. also predicted a sialoside-binding pocket in the NTD of SARS-CoV-2 by surface iso-electron density mapping, further supporting our findings [32]. While the β4-β5 loop is involved in the engagement with all tested sialosides, the β14-β15 loop is specific to larger sialoside such as sLex (Figure 2 and Figure 3). The predicted interacting sites of the tested sialosides are mapped in Figure 3. The presence of key electrostatic and hydrophobic interactions with each of these sialosides suggests the possibility of a physiological interaction with the NTD domain of SARS-CoV-2. The computational binding affinity of the NTD of SARS-CoV-2 with diverse sialosides is compared and analyzed with that of MERS-CoV (Supplementary Figure S2). For both SARS-CoV-2 and MERS-CoV, all sialosides were found to interact and localize to the sialoside-binding pocket in the NTD (Supplementary Figures S2 and S3). These docking results are in strong agreement with the previously published cryo-EM structures of MERS-CoV bound with sialosides (Supplementary Figure S3). [11]. On the other hand, SARS-CoV displayed significant promiscuity and leaky binding with sialosides (Supplementary Figure S2c,d), which was expected since SARS-CoV is not reported to bind with sialosides [11,17]. In particular, Neu5Ac and Neu5Gc occupied different regions within the SARS-CoV NTD suggesting a possible lack of selectivity (Supplementary Figures S2c,d and S3c). Moreover, SARS-CoV displayed lower computational ligand-binding affinities to larger sialosides including 2,3-SLN, 2,6-SLN, and sLeX (Supplementary Figure S2d). Overall, these results indicate the less affinity of SARS-CoV NTD over SARS-CoV-2 and MERS-CoV in engagement with host sialosides.

Molecular dynamics simulation of SARS-CoV-2 NTD-sialoside complexes highlights the flexibility of the β14-β15 loop (Figure 2f). The superimposition of all produced SARS-CoV-2 NTD-sialoside complexes shows an outward movement of the β14-β15 loop, allowing the sialoside-binding site to accommodate larger sialosides such as sLex (Figure 2f and Supplementary Figure S2a). On the other hand, the spike protein of SARS-CoV features a shorter 9 amino acid β14-β15 loop (Figure 1b), which offers reduced degrees of freedom, with a decreased capacity to engage host sialosides (Supplementary Figure S2c,d). In addition, a single-turn alpha helix (Thr20-Leu24) formed key interactions with all sialosides tested (Figure 3). Interestingly, the NTD of MERS-CoV (Gln37-Phe40) [33] and SARS-CoV (Phe22-Val25) also display a single-turn helix suggesting its involvement in sialoside stabilization but not in their recruitment and selectivity (Supplementary Figures S1a and S2). Although HCoV-OC43 spike glycoprotein lacks a single-turn helix, the same region interacts with sialosides [34]. Taken together, these findings suggest that SARS-CoV-2 spike glycoprotein might have independently evolved to recognize sialosides using its longer NTD loop inserts.

In addition to interacting with host protein receptors, the NTD region of coronavirus spike glycoproteins has reciprocally evolved to recognize host sugars, including sialoside receptors [35]. The spike glycoprotein of some beta coronaviruses such as the mouse hepatitis virus interacts exclusively with host protein receptors despite having a sugar-binding pocket in their NTD [35]. Unlike the mouse hepatitis virus, the bovine coronavirus spike glycoprotein does not have a canonical protein receptor and binds solely with 5-N-acetyl-9-O-acetyl neuraminic acid (Neu5,9Ac2) to infect host cells [31,33]. Among human-infecting coronaviruses, some bind exclusively to host sialosides like HCoV-OC43 and HCoV-229E, whereas others like SARS-CoV utilize only protein receptors to infect host cells. Recent studies suggest that MERS-CoV utilizes a dual-receptor mechanism to infect host cells, where it binds with both human dipeptidyl peptidase-4 (DPP4) host protein receptor and host sialosides [11]. Such unique evolutionary co-adaptation to amalgamate the recognition of both protein and sialoside receptors provide an additional bridging mechanism for effective virus attachment, cellular entry, and tropism of the virus.

Thus, the acquired ability of SARS-CoV-2 to engage diverse sialosides as reciprocal host-cell receptors might be the reason for its high infectivity with broad tissue tropism. Taken together, the differential distribution of sialosides in the respiratory tract and other organs, along with limited ACE2 expression in human airway epithelia [9] would explain differential infectivity, transmission, and tropism shown by SARS-CoV-2 [36]. In connotation to this, the recent preprint report suggests that the SARS-CoV-2 spike glycoprotein recognizes different siglecs (sialic acid-binding Ig-like lectins) and C-type lectins; suggesting that spike glycoprotein interacts in ACE2-independent infection pathways with the immune cells [37]. Besides, the human ABO blood group and COVID-19 susceptibility [38,39] may relate to modulation of the sialosides distribution pattern on the target membrane, possibly regulating SARS-CoV-2 transmission and tropism [40] despite high affinity with ACE2. When confirmed by biophysical and structural binding studies, the in silico structural analysis reported in this study will provide a basis for further research to explore the functional role of the reciprocal interaction of SARS-CoV-2 with host sialic acid during virus entry and spread.

## Supplementary Materials

The following are available online at https://www.mdpi.com/1999-4915/12/9/909/s1: Figure S1. Superimposition of SARS-CoV-2 NTD with MERS-CoV NTD and HCoV-OC43 NTD. Figure S2. Comparative binding analysis of sialosides with the NTD of SARS-CoV-2, MERS-CoV and SARS-CoV spike glycoproteins. Figure S3. Comparative binding analysis of Neu5Ac with the NTD of SARS-CoV-2, MERS-CoV and SARS-CoV spike glycoproteins. Table S1. Stereochemical validation statistics for the full-length model of SARS-CoV-2, MERS-CoV and SARS-CoV spike glycoproteins.

## Abbreviations

NTD	N-terminal domain
SARS	Severe acquired respiratory syndrome
MERS	Middle East respiratory syndrome
Neu5Ac	5-N-acetyl neuraminic acid
Neu5Gc	5-N-glycolyl neuraminic acid
