# Supplementary material for: The Sialoside-Binding Pocket of SARS-CoV-2 Spike Glycoprotein Structurally Resembles MERS-CoV
Source: Viruses. 2020 Aug 19;12(9):909. doi: 10.3390/v12090909 (PMC7551769; doi:10.3390/v12090909)
Supplement: Supplementary file 1 [file viruses-12-00909-s001.pdf]

viruses

MDPI

Supplementary Materials

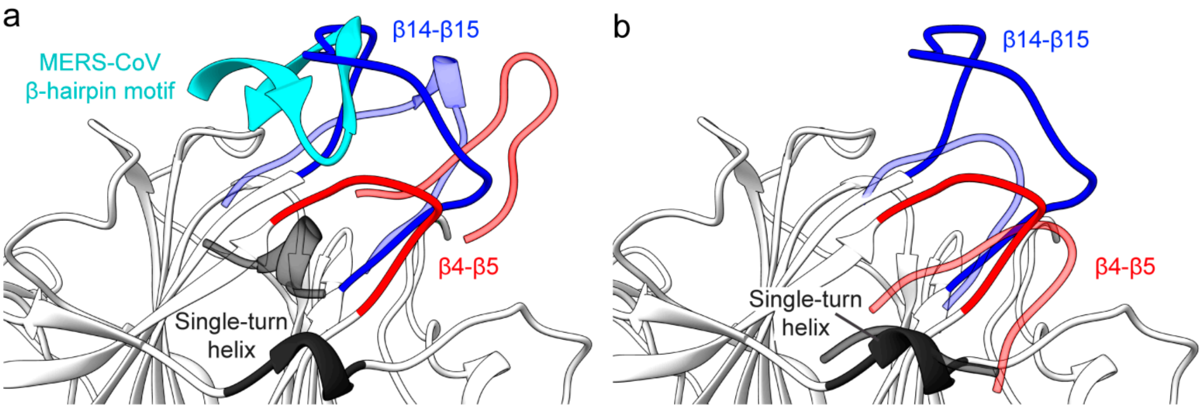

Supplementary Figure 1. Superimposition of SARS-CoV-2 NTD with MERS-CoV NTD (**a**) and HCoV-OC43 NTD (**b**) showing the relative arrangement of divergent loop regions (β4-β5, red; β14-β15, blue) and other key components (single-turn helix, black; MERS-CoV β-hairpin motif, teal) that participate in sialoside binding. The rest of the MERS-CoV NTD and HCoV-OC43 NTD is not displayed for clarity. Loop regions with increased transparency belong to either MERS-CoV (**a**) or HCoV-OC43 (**b**).

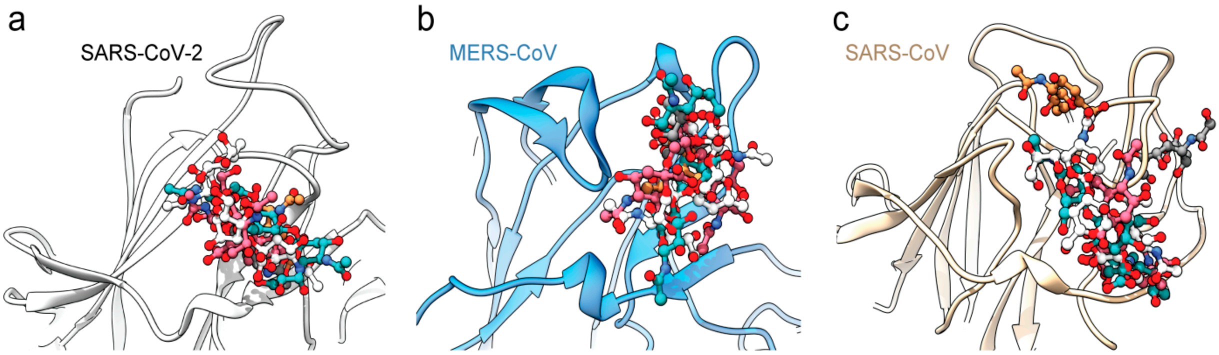

d

	Neu5Ac	2,3-SLN	2,6-SLN	Neu5Gc	sLex
SARS-CoV-2 binding affinity (kcal/mol)	-6.1	-6.5	-5.8	-6.4	-6.2
MERS-CoV binding affinity (kcal/mol)	-5.5	-5.3	-5.7	-5.0	-4.7
SARS-CoV binding affinity (kcal/mol)	-5.6	-4.6	-3.6	-5.1	-2.7

Supplementary Figure 2. Comparative binding analysis of sialosides with the NTD of SARS-CoV-2, MERS-CoV and SARS-CoV spike glycoproteins. All tested sialosides bind specifically to SARS-CoV-2 (**a**) and MERS-CoV (**b**) sialoside-binding pocket. (**c**) Diverse sialosides occupy different regions within the NTD of SARS-CoV suggesting non-specific interactions. (**d**) Computational binding affinities of the SARS-CoV-2, MERS-CoV and SARS-CoV spike glycoproteins with sialosides.

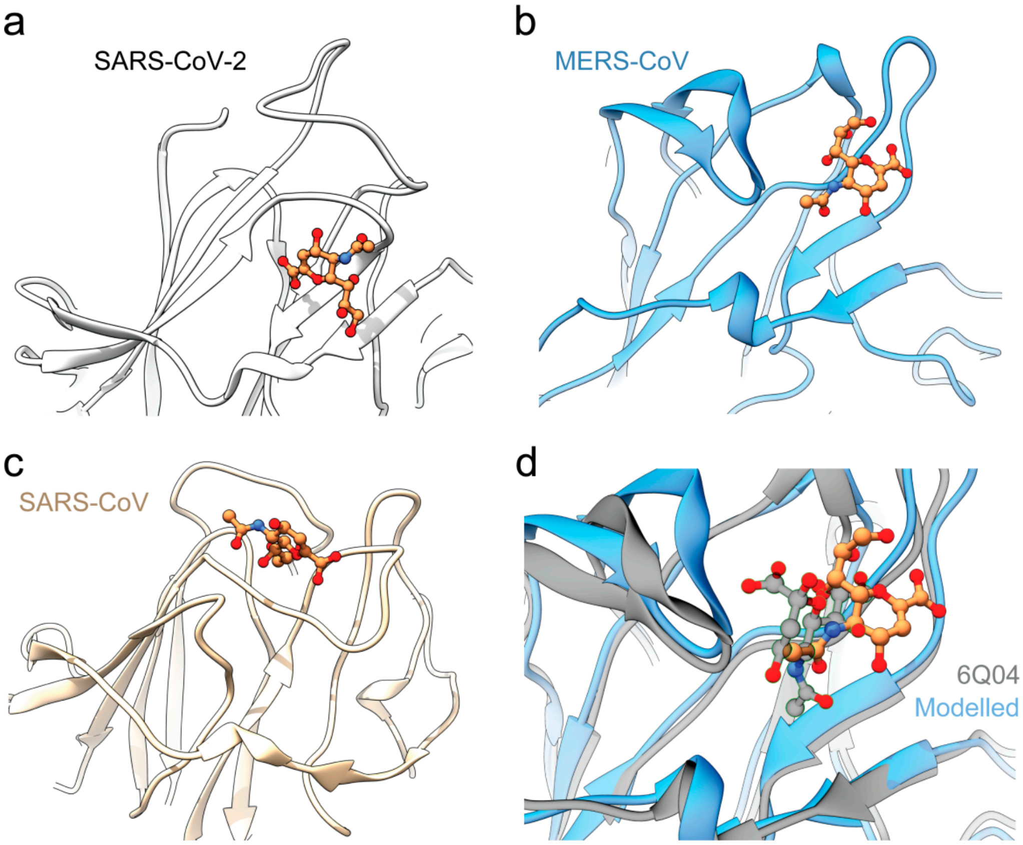

Supplementary Figure 3. Comparative binding analysis of Neu5Ac with the NTD of SARS-CoV-2 (**a**), MERS-CoV (**b**) and SARS-CoV (**c**) spike glycoproteins. (**d**) Overlay of the cryo-EM structure of MERS-CoV bound with Neu5Ac (grey) with the modelled MERS-CoV NTD (blue ribbon) docked with Neu5Ac (orange).

Supplementary Table 1. Stereochemical validation statistics for the full-length model of SARS-CoV-2, MERS-CoV and SARS-CoV spike glycoproteins.

	**SARS-CoV-2**	**MERS-CoV**	**SARS-CoV**
**Structure evaluation**			
Clashscore	4.10	5.48	2.66
Ramachandran favored (%)	90.69	86.28	92.58
Ramachandran outliers (%)	0	0	0
Cβ deviations >0.25Å (%)	0	0	0
Bad bonds (%)	0	0	0
Bad angles (%)	0	0	0
Cis Prolines (%)	0	0	0
Percentile	96th	92nd	98th

Stereochemical qualities of all the protein models were assessed with the Molprobity server.
